# ESPO and Social Research, Policy, and Practice Section Symposium: Innovative Translational Research to Improve Home Environment and Activity Performance for Aging in Place

**DOI:** 10.1093/geroni/igaf122.333

**Published:** 2025-12-31

**Authors:** Mengzhao Yan, Taylor Jansen, Sarah Szanton

## Abstract

Ensuring home safety and accessibility is essential for aging in place, yet the diverse needs of older adults demand innovative and person-centered approaches. The 2025 GSA SRPP ESPO Symposium brings together a panel of established transdisciplinary gerontology researchers with training backgrounds in multiple disciplines. They will share insights from their multidimensional careers and highlight their innovative research projects that translate science and technologies into practical applications, devices, and programs to improve the home environment and activity performance for older adults. Professor Wendy Rogers of the University of Illinois Urbana-Champaign will introduce her work at the Human Factors and Aging Laboratory and the McKechnie Family LIFE Home to illuminate human-technology interaction. Professor Susan Stark of the Washington University in Saint Louis will focus on the Home Hazard Removal Program (HARP) to illustrate collaborative efforts and strategies to develop and deliver interventions. Professor Laura Gitlin of Drexel University will elucidate the promise and future of intervention science, using the Care of Older Persons in their Environment (COPE) program and similar programs as exemplars. Professor Jon Sanford of Georgia State University will present his career in evidence-based, user-centered design. Professor Sarah Szanton of Johns Hopkins University, Co-Developer of the Community Aging in Place Advancing Better Living for Elders (CAPABLE) program, will serve as the discussant and lead a discussion aimed at offering advice for emerging scholars and professionals on integrating knowledge across disciplines, driving innovations to advance gerontology, and bridging research, policy, and practice to promote person-centered approaches for aging in place.

